# A toolbox for brain network construction and classification (BrainNetClass)

**DOI:** 10.1002/hbm.24979

**Published:** 2020-03-12

**Authors:** Zhen Zhou, Xiaobo Chen, Yu Zhang, Dan Hu, Lishan Qiao, Renping Yu, Pew‐Thian Yap, Gang Pan, Han Zhang, Dinggang Shen

**Affiliations:** ^1^ College of Computer Science and Technology Zhejiang University Hangzhou China; ^2^ Department of Radiology and BRIC University of North Carolina at Chapel Hill Chapel Hill North Carolina USA; ^3^ Automotive Engineering Research Institute Jiangsu University Zhenjiang China; ^4^ Department of Psychiatry and Behavior Sciences Stanford University Stanford California USA; ^5^ School of Mathematics Science Liaocheng University Liaocheng China; ^6^ School of Electric Engineering Zhengzhou University Zhengzhou China

**Keywords:** brain connectome, dynamic functional connectivity, functional connectivity, machine learning, prediction, sparse representation, toolbox

## Abstract

Brain functional network has been increasingly used in understanding brain functions and diseases. While many network construction methods have been proposed, the progress in the field still largely relies on static pairwise Pearson's correlation‐based functional network and group‐level comparisons. We introduce a “Brain Network Construction and Classification (BrainNetClass)” toolbox to promote more advanced brain network construction methods to the filed, including some state‐of‐the‐art methods that were recently developed to capture complex and high‐order interactions among brain regions. The toolbox also integrates a well‐accepted and rigorous classification framework based on brain connectome features toward individualized disease diagnosis in a hope that the advanced network modeling could boost the subsequent classification. BrainNetClass is a MATLAB‐based, open‐source, cross‐platform toolbox with both graphical user‐friendly interfaces and a command line mode targeting cognitive neuroscientists and clinicians for promoting reliability, reproducibility, and interpretability of connectome‐based, computer‐aided diagnosis. It generates abundant classification‐related results from network presentations to contributing features that have been largely ignored by most studies to grant users the ability of evaluating the disease diagnostic model and its robustness and generalizability. We demonstrate the effectiveness of the toolbox on real resting‐state functional MRI datasets. BrainNetClass (v1.0) is available at https://github.com/zzstefan/BrainNetClass.

## 1 INTRODUCTION

Functional connectivity (FC) based on resting‐state functional MRI (rs‐fMRI) is one of the major methods for brain functional studies. It describes functional interactions among anatomically separated brain regions, interpreted as information exchange, a.k.a. functional integration (Allen et al., 2014; Hutchison et al., 2013; Leonardi et al., 2013; Thomas Yeo et al., 2011; Van Dijk et al., 2009). Whole‐brain large‐scale FC network is believed to be essential neural substrates for complex cognitive functions (Jiang et al., 2019; Li et al., 2020; Wen et al., 2019; Zhou et al., 2019), usually modeled as a graph, with nodes representing brain regions and edges inter‐regional FCs (Hallquist & Hillary, 2018; Sporns, 2010; Van Den Heuvel & Pol, 2010). The network topological properties can be analyzed based on statistical or computational methods such as graph theoretical analysis (Bullmore & Bassett, 2011; Li et al., 2014) to reveal disease‐related alterations (Badhwar et al., 2017; Fornito, Zalesky, & Breakspear, 2015).

While many studies focused on group‐level differences in the brain functional networks between patients and healthy controls based on statistical inference, an emerging trend is to utilize machine learning techniques to learn diagnostic connectomic patterns and conduct individualized classification for computer‐aided diagnosis (Arbabshirani, Plis, Sui, & Calhoun, 2017; Dubois & Adolphs, 2016; Rathore, Habes, Iftikhar, Shacklett, & Davatzikos, 2017). It is highly desirable by clinicians to identify diseased subjects (Shin et al., 2016; Tan et al., 2019), plan personalized treatment (Gudayol‐Ferré, Peró‐Cebollero, González‐Garrido, & Guàrdia‐Olmos, 2015; Miao et al., 2017; Miao et al., 2018), or predict outcome of medication (Fan et al., 2007; Liu et al., 2018; Nie et al., 2019). From the methodological point of view, such a brain functional network‐based classification is essentially a pattern recognition problem, where discriminative features (e.g., FC links or network properties) can be jointly learned from the brain networks and weighted in a multivariate manner toward a classification outcome. It not only helps with better patient‐control separation but also benefits imaging biomarker detection for better understanding brain diseases (Jie et al., 2013; Yu et al., 2017; Zhang, Zhang, et al., 2019).

With the fast development in both brain network modeling (Calhoun, Miller, Pearlson, & Adali, 2014; Dadi et al., 2019; Smith et al., 2011) and machine learning methods (Bishop, 2006; Xu, Qi, et al., 2018), rs‐fMRI‐based clinical studies have been transforming from bench‐ to bed‐side at an unprecedented speed (Cui & Gong, 2018; Lemm, Blankertz, Dickhaus, & Müller, 2011; Pereira, Mitchell, & Botvinick, 2009; Shen, Wu, & Suk, 2017). However, the broad interests are not accompanied by sufficient analytic tools for researchers from multiple disciplines to conduct brain network construction and network‐based classification. On one hand, the majority of the brain FC network studies still largely relies on the traditional, static, pairwise Pearson's correlation‐based functional network construction. Disease diagnosis could largely benefit from advanced brain functional network construction methods that could model high‐level, more complex brain functional interactions among multiple brain regions, which might be more sensitive to the disease‐related alterations. On the other hand, neuroscientists and clinicians with their respective abundant domain knowledge are in a pressing need of such multivariate analysis‐derived biomarkers but not always equipped with the same amount of knowledge on imaging analysis, network construction, and machine learning. Without the help of rigorously designed toolboxes, they might face problems such as double dipping (training and testing a classification model with the same data) (Kriegeskorte, Simmons, Bellgowan, & Baker, 2009). Moreover, for classifiers with freely estimable parameters, arbitrary parameter predefinition, or ad hoc parameter selection is not uncommon (Demirci et al., 2008). All these issues harm the generalization ability of the diagnostic model, leading to degraded reproducibility and finally hindering clinical applications. A toolbox with standardized and rigorous network‐based classification is highly demanded (Cui & Gong, 2018).

In this article, we present a novel toolbox, namely Brain Network Construction and Classification toolbox (BrainNetClass, currently in v1.1). It is a user‐friendly graphical‐user‐interface (GUI)‐based Matlab toolbox designed to help neuroscientists, doctors, and researchers in other fields easily and rigorously work on advanced brain functional connectomics construction and connectomics‐based individualized disease diagnosis or other classification tasks. It avoids complicated demand to the end users by providing them with an easy‐to‐use, automated pipelined toolbox that turns BOLD (blood oxygen level dependent) rs‐fMRI time series into brain functional networks with advanced methods and generates a strictly‐designed, well‐accepted classifier for disease diagnosis. It also produces comprehensive and interpretable results for model evaluation toward better understanding of brain diseases. This toolbox was designed to help with standardizing methodology and boosting reproducibility, generalizability, and interpretability of the brain network‐based classification.

BrainNetClass features the following advantages compared to most of the existing toolboxes. (a) It provides state‐of‐the‐art algorithms for brain network constructions ranging from a recently developed high‐order functional network for quantifying higher level FC that reflects more complex brain functional organization principals (Zhang, Chen, Zhang, & Shen, 2017) to a sparse representation‐based brain network construction that generates more robust and biologically meaningful networks. (b) It is pipelined, automated, together with parameter optimization through nested cross‐validation (note that GraphVar is also pipelined and with parameter optimization for the classifiers). (c) It offers a comprehensive battery of result evaluation metrics, including some usual analyses, such as parameter sensitivity test, model robustness test (GraphVar also includes a bootstrapping‐based robustness test), discriminative features, among many others. (d) It is flexible, allowing users to use command line mode or GUI mode and to save the constructed brain networks for other purposes. Generally, BrainNetClass finds it seats between widely adopted rs‐fMRI preprocessing software (DPABI (Yan, Wang, Zuo, & Zang, 2016), Brant (Xu, Liu, Zhan, Ren & Jiang, 2018), and SPM^1^) and many other Matlab‐based network‐based post‐analysis toolkits (Brain Connectivity Toolbox (Rubinov & Sporns, 2010), CONN (Whitfield‐Gabrieli & Nieto‐Castanon, 2012), and GraphVar (Kruschwitz, List, Waller, Rubinov, & Walter, 2015; Waller et al., 2018)), warranting its necessity and practicability.

In the following sections, we introduce all the involved advanced brain functional network modeling methods in Section 2.1 and a brief classification procedure in Section 2.2, by highlighting the innovative and comprehensive result report in Section 2.3. After brief descriptions of the toolbox modules, a walk‐through, and the advanced commands and flexible I/O in Sections 3.1–3.3, we demonstrate four real applications with different classification goals in Section 4. We finish up with key discussions on practical guidance and other aspects.

## 2 MATERIALS AND METHODS

### 2.1 Functional network construction

Pearson's correlation (PC) analysis between BOLD rs‐fMRI signals associated with any pair of ROIs is the most popular FC network construction method. PC is intuitive and easy to interpret, but only captures the pairwise relationship between two ROIs. Partial correlation, or more generally, sparse representation (SR) is a popular method to characterize multi‐ROI relationship. In SR, the BOLD signal of a brain region is represented by a linear combination of the signals from a few of other regions. To measure higher‐level relationship between two ROIs, “high‐order” FC (HOFC) was proposed to define inter‐regional relationship by not measuring “low‐level” features (i.e., BOLD signals) but various “high‐level” features, which provides complementary information to the traditional “low‐order” (PC‐based) brain networks and indicates improved performance in disease diagnosis (Zhang, Chen, et al., 2016; Zhang, Chen, et al., 2017; Zhang, Shen, & Lin, 2019; Zhou, Zhang, Teng, Qiao, & Shen, 2018; Zhou, Qiao, Li, Zhang, & Shen, 2018). On the other hand, to avoid too sparse (thus may miss disease‐related FC alterations) brain networks derived from SR and respect inherent structures in the brain network, recent research has been designing new regularization terms to build more biologically meaningful brain networks, resulting in many variants of the SR methods (Qiao et al., 2016; Yu et al., 2017; Zhang, Zhang, et al., 2019). While promising, these novel methods require more complex computations than PC does.

BrainNetClass enables users to implement these state‐of‐the‐art network construction methods to bring the methodological advance to the connectome‐based disease studies. In next paragraphs, we will provide a brief introduction of each network construction method; for more details, refer to respective original methodology papers. We tentatively categorize these algorithms into two types, pairwise and multi‐ROI‐based (or SR‐based) methods. In the toolbox GUI, they are grouped into methods without any need of parameter optimization and methods requiring parameter optimization, an implementation‐orientated categorization. Table 1 summarizes the meaning of the symbols used later. Note that there are many other advanced network construction methods such as brain states analysis based on dynamic FC (Calhoun et al., 2014), but it is not the topic of this article and not included in BrainNetClass.

**Table 1:** Symbols used in the current study

Symbol	Meaning
*L*	Window length of sliding windows for dynamic FC
*M*	Number of subjects
*N*	Number of brain ROIs
*T*	Number of time points in rs‐fMRI data
*K*	Number of clusters (new nodes) for clustering dynamic FC time series
**W**	Connectivity matrix, or brain functional network
*w*_*ij*_	FC weight of an edge connecting two nodes (*i*, *j*) in a network **W**
**X**,**X**^*m*^	rs‐fMRI data matrix of the *m*th subject
*x*_*i*_	Mean rs‐fMRI time series of the *i*th ROI

Abbreviation: FC, functional connectivity.

#### 2.1.1 Pairwise FC‐based network construction methods

Given a brain parcellation atlas with *N* ROIs, rs‐fMRI signal at the *i*th ROI can be represented as a column vector *x*_*i*_ = [*x*_1*i*_, *x*_2*i*_, …, *x*_*Ti*_]^′^ ∈ ℝ^*T*^ (′ denotes transpose operation), and a data matrix **X** = [*x*_1_, *x*_2_, …, *x*_*N*_] ∈ ℝ^*T* × *N*^. PC‐based brain functional network can be represented as a graph with an edge weight matrix **W** ∈ ℝ^*N* × *N*^ whose element *w* is calculated by pairwise temporal correlation of the raw BOLD signals. In BrainNetClass, PC‐derived FC network usually serves as a baseline method to be compared with other advanced methods. To differentiate from the high‐order FC methods (HOFC) (Jia, Zhang, Adeli & Shen, 2017; Zhang, Zhang, Chen, & Shen, 2017; Chen, Zhang, & Shen, 2016), it is also referred to as low‐order FC (LOFC).

In a similar manner, with each ROI's (one‐to‐all) topographical FC profiles used as high‐level features of this ROI, topographical profile similarity‐based HOFC (tHOFC) can be calculated using PC on the features between each pair of ROIs:

(1) $${\text{tHOFC}}_{\mathrm{ij}}=\frac{\sum_{k}\left(w_{\mathrm{ik}}-\bar{w_{i\cdot }}\right)\left(w_{\mathrm{jk}}-\bar{w_{j\cdot }}\right)}{\sqrt{\sum_{k}{\left(w_{\mathrm{ik}}-\bar{w_{i\cdot }}\right)}^{2}}\sqrt{\sum_{k}{\left(w_{\mathrm{jk}}-\bar{w_{j\cdot }}\right)}^{2}}}$$

where *w*_*i*·_ = {*w*_*ik* ∣ *k* ∈ *N*, *k* ≠ *i*_} and *i*, *j*, *k* = 1, 2, …, *N*, *k* ≠ *i*, *j*. Due to the LOFC features rather than the BOLD signals are used in the tHOFC calculation, the result is essentially different from that of LOFC between the same ROIs. It has been shown that tHOFC could provide supplementary information to the conventional LOFC and help revealing additional group differences between mild cognitive impairment (MCI) subjects and cognitively normal controls (Zhang, Chen, et al., 2016; Zhang, Giannakopoulos, et al., 2019).

Associated HOFC (aHOFC) is defined based on pairwise PC between the topographical profiles of tHOFC and those of LOFC for any pair of ROIs, in a similar manner as PC and tHOFC (Equation (2)). In the psychophysiological interaction modeling, high‐level preset of a psychological statuses (e.g., attention level) may change sensory information collection, processing, and synthesis. Similarly, in the human brain network, different brain regions collaborate with each other at different levels, mediating different sensory and cognitive functions. For example, the LOFC may collect and process domain‐specific information while the tHOFC may further integrate the information from multiple domains according to the functional hierarchy. aHOFC can measure such inter‐level (between low‐level and high‐level) functional associations and complements the LOFC and tHOFC. Including all three types of the pairwise functional association indices (namely, hybrid HOFC) could further improve the diagnostic accuracy of MCI diagnosis (Zhang, Zhang, Chen, Lee, & Shen, 2017). By definition, aHOFC matrix is not symmetric, and the self‐connections are not 1 s, which is unlike PC and tHOFC. We found that the upper triangular and the lower triangular of the aHOFC matrices are highly correlated. Therefore, the matrix symmetry should have very limited influence on the result. In practice, to make the generated matrix more interpretable, we further symmetrized it by **W** ← (**W** + **W**^′^)/2.

(2) $${\text{aHOFC}}_{\mathrm{ij}}=\frac{\sum_{k}\left({\text{tHOFC}}_{\mathrm{ik}}-\bar{{\text{tHOFC}}_{i\cdot }}\right)\left(w_{\mathrm{jk}}-\bar{w_{j\cdot }}\right)}{\sqrt{\sum_{k}{\left({\text{tHOFC}}_{\mathrm{ik}}-\bar{{\text{tHOFC}}_{i\cdot }}\right)}^{2}}\sqrt{\sum_{k}{\left(w_{\mathrm{jk}}-\bar{w_{j\cdot }}\right)}^{2}}}$$

Increasing evidence has shown that FC is actually varying across time and such variation could not be purely caused by noise. Such a dynamic FC may reflect brain flexibility and moment‐to‐moment adaption (Gonzalez‐Castillo et al., 2015). Chen et al. (2016) proposed a new brain network construction method based on dynamic FC, namely dynamics‐based HOFC (dHOFC). First, dynamic FC *w*_*ij*_(*θ*) is calculated between ROIs *i* and *j* based on BOLD rs‐fMRI signals in the sliding windows (*θ* = 1, 2, …, Θ), each of which includes a small temporal segment of rs‐fMRI signals in a length of *L* with a step size *s* (thus, Θ = ⌊(*T* − *L*)/*s*⌋ + 1). Then, dHOFC is calculated based on another round of PC between any pair of the dynamic FC time series (Equation (3)).

(3) $${\text{dHOFC}}_{\mathrm{ij},\mathrm{pq}}=\frac{\sum_{\theta =1}^{\Theta }\left(w_{\mathrm{ij}}\left(\theta \right)-\bar{w_{\mathrm{ij}}\left(\cdot \right)}\right)\left(w_{\mathrm{pq}}\left(\theta \right)-\bar{w_{\mathrm{pq}}\left(\cdot \right)}\right)}{\sqrt{\sum_{\theta =1}^{\Theta }{\left(w_{\mathrm{ij}}\left(\theta \right)-\bar{w_{\mathrm{ij}}\left(\cdot \right)}\right)}^{2}}\sqrt{\sum_{\theta =1}^{\Theta }{\left(w_{\mathrm{pq}}\left(\theta \right)-\bar{w_{\mathrm{pq}}\left(\cdot \right)}\right)}^{2}}}$$

By definition, dHOFC_*ij*, *pq*_ characterizes temporal synchronization of dynamic FC time series, thus defining the relationship among four, instead of two ROIs. Therefore, the dHOFC network is defined in a ${\mathbb{R}}^{N^{2}\times N^{2}}$ space, instead of PC, tHOFC, and aHOFC networks (all in a ℝ^*N* × *N*^ space). To reduce the exponentially increased dimensionality for better classification, the third step of dHOFC is to run a clustering algorithm to reduce the dimension from *N*^2^ × *N*^2^ to *K* × *K*, where *K* is the number of clusters, or “high‐order nodes” in the dHOFC network. By grouping the dynamic FC time series with similar temporal patterns, we use the cluster averaged dynamic FC time series to construct a lower‐dimension approximation of the dHOFC ∈ ℝ^*K* × *K*^. The window length *L* and the cluster number *K* are two important parameters for dHOFC.

#### 2.1.2 Multi‐region‐based network construction methods

The methods in Section 2.1.1 all measures pairwise relationship, in this section, we introduce SR‐based methods for measuring inter‐regional relationship when considering other regions' influence. The SR‐based methods also have an advantage that it could suppress spurious connections as shown in PC‐based results. SR generates the network **W** by adding a *l*_1_‐norm regularization in a matrix inversion problem to reveal two regions' relationship after ruling out the influence from other regions, efficiently conducting the partial correlation between any pair of ROIs. It does so by minimizing the loss function denoted in Equation (4):

(4) $$\min_{W}\frac{1}{2}{\left\|X-\mathrm{XW}\right\|}_{F}^{2}+\lambda {\left\|W\right\|}_{1}$$

where *λ* > 0 is a parameter controlling network sparsity. A higher *λ* forces more links in **W** to be zeros (no connection). In the toolbox, for all the SR‐based methods (including SR), an additional step is conducted to make the network symmetric, similar to that used in aHOFC via **W** ← (**W** + **W**^′^)/2 (Yu et al., 2019; Zhang, Zhang, et al., 2019). Of note, another symmetrization method $w_{\mathrm{ij}}^{*}\leftarrow \mathrm{sign}\left(w_{\mathrm{ij}}\right)\sqrt{w_{\mathrm{ij}}w_{\mathrm{ji}}}$ can also be used (Peng, Wang, Zhou, & Zhu, 2009). The SR can serve as another baseline method. Next, we introduce several SR variations, which makes the resultant networks have additional desired properties (e.g., modular structure). Of note, we use SLEP (Liu, Ji, Ye, et al., 2009) package v4.1^2^ for optimization for all SR‐related methods.

An FC‐strength penalty was introduced in SR, namely weighted sparse representation (WSR) (Yu et al., 2017; Chen, Zhang, Zhang, Li, & Shen, 2017). In WSR, the sparse regularization is weighted by $C_{\mathrm{ij}}=\exp\left(-{\mathrm{FC}}_{\mathrm{ij}}^{2}/\sigma \right)$, where the FC_*ij*_ is the PC‐based FC strength between the *i*th and *j*th ROIs and *σ* is a positive parameter (*σ* can be set as all subjects' mean of standard variation of the absolute PC‐based FC strengths) used to adjust the decay speed of the FC‐based weights (Equation (5)). If the BOLD signals of two ROIs strongly synchronized (indicating a strong FC), then their connection should be penalized less, thus making it more possible to be retained to preserve potentially biologically putative FC links. It has been shown that the WSR network is more biologically meaningful and more suitable for disease diagnosis than the SR network (Yu et al., 2017).

(5) $$\min_{W}\frac{1}{2}{\left\|X-\mathrm{XW}\right\|}_{F}^{2}+\lambda_{1}{\left\|C⊙W\right\|}_{1}$$

In another method called strength‐weighted sparse group representation (WSGR), strong FC links can be well preserved as in WSR, and the original structured FC information in the PC‐derived network can also be preserved, thanks to another regularization term encouraging a joint preservation or suppression of a group of FC links with similar strength (Simon, Friedman, Hastie, & Tibshirani, 2013; Yu et al., 2017). In WSGR, the PC‐derived FC links are first grouped into a few subsets {*O*_*g*_, *g* = 1, 2, …, *G* (*G* ≪ *N*)}, each of which is assigned a predefined weight $d_{g}=\exp\left(-E_{g}^{2}/\sigma \right)$, where *E*_*g*_ is the averaged absolute PC‐based FC strength for the subset *O*_*g*_ and *σ* is set as all subjects' mean of standard variation of absolute PC‐based FC strengths. Then, the WSGR can be formatted as Equation (6), where ${\left\|W_{O_{g}}\right\|}_{2}=\sqrt{\sum_{\left(i,j\right)\in O_{g}}{\left(w_{\mathrm{ij}}\right)}^{2}}$ is a *l*_2_‐norm penalty for each subset *O*_*g*_ for joint selection or de‐selection. Collectively, WSGR results in an FC network featuring overall sparsity (controlled by *l*_1_‐norm penalty), and group sparsity (controlled by *l*_2_‐norm penalty). WSGR has two parameters (*λ*_1_ and *λ*_2_) to optimize for balancing such a tradeoff.

(6) $$\min_{W}\frac{1}{2}{\left\|X-\mathrm{XW}\right\|}_{F}^{2}+\lambda_{1}{\left\|C⊙W\right\|}_{1}+\lambda_{2}\sum_{g=1}^{G}d_{g}{\left\|W_{O_{g}}\right\|}_{2}$$

It is notable that the SR constructs a network for each subject independently. This could lead to large inter‐subject variability in the derived networks. This is unfavorable for subsequent classification, as it will increase within‐group variability and make between‐group separation more difficult. Group sparse representation (GSR) is put forward to address this problem by jointly estimating non‐zero connections across all subjects (Wee, Yap, Zhang, Wang, & Shen, 2014; Zhang, Zhang, Chen, Liu, Zhu, & Shen, 2017). It encourages the derived connectivity networks to have similar topological structures across all the subjects through a *l*_2, 1_‐norm regularizer, as formulated in Equation (7), where $W_{i}=\left[w_{i}^{1},\ldots ,w_{i}^{m},\ldots ,w_{i}^{M}\right]$ denotes the regional one‐to‐all PC‐derived FC profiles of the *i*th ROI for all *M* subjects and *λ* controls the extent of group sparsity.

(7) $$\min_{W_{i}}\sum_{m=1}^{M}\left(\frac{1}{2}{\left\|x_{i}^{m}-X_{i}^{m}w_{i}^{m}\right\|}_{2}^{2}\right)+\lambda {\left\|W_{i}\right\|}_{2,1}$$

Zhang, Zhang, et al. (2019) recently proposed another GSR, namely strength and similarity guided GSR (SSGSR), aiming at better separating two groups. They assumed that the PC‐derived FC networks should inherently have higher within‐group similarity but lower between‐group similarity. For example, a network from a patient could be more similar to that from another patient but less similar to that from a healthy control. To this end, the inter‐subject similarity of the PC‐derived FC profiles can be used as a regularizer as the last term of Equation (8). Letting $w_{i.}^{m}$ and $w_{i.}^{l}$ be the regional one‐to‐all PC‐derived FC profiles of the *i*th ROI from the *m*th subject and the *l*th subject, a graph Laplacian ℒ_*i*_ can be obtained by ℒ_*i*_ = **D**_*i*_ − **S**_*i*_, where $S_{i}=\left\{s_{i}^{m,l}\right\}\in {\mathbb{R}}^{N\times N}$ is a similarity matrix with element $s_{i}^{m,l}=\exp\left(-{\left\|w_{i.}^{m}-w_{i.}^{l}\right\|}_{2}^{2}\right)$ measuring the FC‐profile similarity for the *i*th ROI between the two subjects.

(8) $$\min_{W_{i}}\sum_{m=1}^{M}\left(\frac{1}{2}{\left\|x_{i}^{m}-X_{i}^{m}w_{i}^{m}\right\|}_{2}^{2}\right)+\lambda_{1}{\left\|B_{i}⊙W_{i}\right\|}_{2,1}+\lambda_{2}\mathrm{tr}\left(W_{i}{ℒ}_{i}{\left(W_{i}\right)}^{T}\right)$$

The second term of Equation (8) has a weight matrix $B_{i}=\left[b_{i}^{1},\ldots ,b_{i}^{m},\ldots ,b_{i}^{M}\right]$ with each column characterizing exponentially‐transformed one‐to‐all PC‐derived FC profiles $\left\{b_{i,j}^{m}=\exp\left(-{\left(w_{i,j}^{m}\right)}^{2}\right)\right\}\left(i,j=1,\ldots ,N,i\neq j\right)$ to penalize weak connectivity. There are two parameters controlling the tradeoff between weighted group sparsity (*λ*_1_) and between‐subject variability (*λ*_2_). Of note, SSGSR (including GSR) were designed to construct brain networks for all of the subjects simultaneously by leveraging the group information to ensure the networks constructed are consistent across individuals. The subject labels will not be used when constructing the brain networks.

Qiao et al. (2016) and Qiao, Zhang, Chen, and Shen (2018) proposed another functional brain network construction method, namely sparse low‐rank (SLR) graph learning, by incorporating a low‐rank prior into the SR‐based network modeling. SLR results in a sparse yet modularity structure‐preserved (more and stronger within‐module connections but less and weaker between‐module connections) FC network, which is considered biologically meaningful (Bullmore & Sporns, 2012; Newman, 2006). It has been shown that, by increasing modularity of the constructed FC network, disease classification accuracy could be increased (Qiao et al., 2016). SLR is formulated in Equation (9), where ‖**W**‖_*_ is a trace norm (a.k.a. nuclear norm) that encourages the estimated adjacency matrix **W** to have a low‐rank property. The combination of sparse and low‐rank properties mathematically lead to a larger modularity. It has two parameters (*λ*_1_, controlling sparsity, and *λ*_2_, controlling modularity) to be optimized.

(9) $$\min_{W}{\left\|X-\mathrm{XW}\right\|}_{F}^{2}+\lambda_{1}{\left\|W\right\|}_{1}+\lambda_{2}{\left\|W\right\|}_{*}$$

### 2.2 Network‐based classification

**Figure 1:**
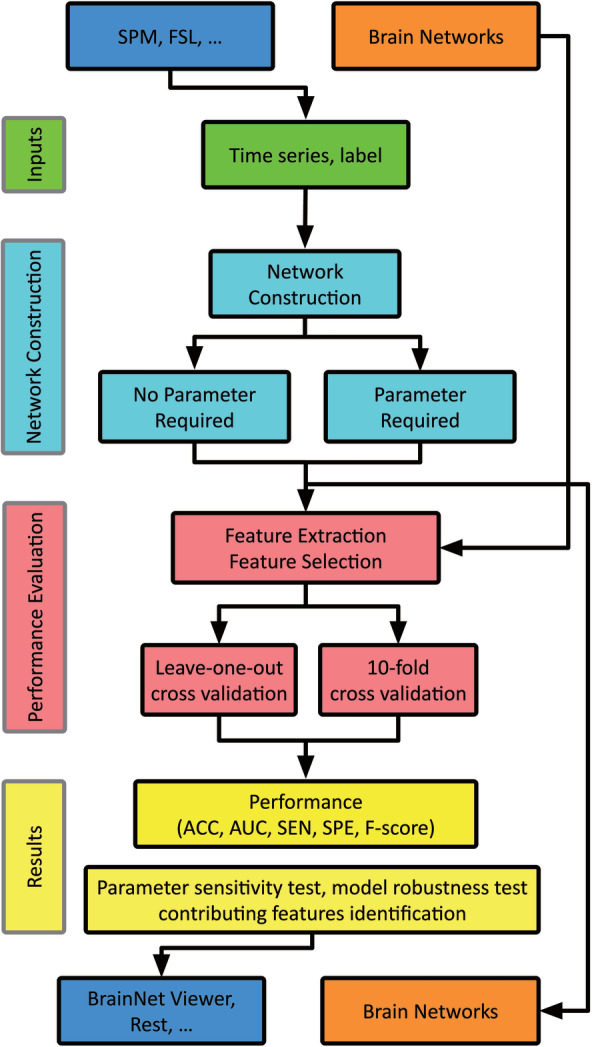
Workflow of BrainNetClass toolbox and its role in a general setting. BrainNetClass receives inputs from many other rs‐fMRI preprocessing software, performs network construction, and extracts network features for classification. After performance evaluation, results are saved for further interpretation and they can be visualized by other toolboxes. It also has flexibility if users just want to save the constructed networks for another purpose or they have predefined brain networks derived from another toolbox

After brain network is constructed, BrainNetClass continues to conduct feature extraction from the constructed networks and train a classifier. Figure 1 summarizes the workflow. As the main contribution of BrainNetClass is providing advanced network construction methods (Section 2.1) and comprehensive results (Section 2.3), we only briefly describe the classification (more details can be found elsewhere).

As for features, both connection coefficients and network property for each ROI (local clustering coefficients (Rubinov & Sporns, 2010; Chen et al., 2016; Zhang, Zhang, et al., 2017) is provided in this version) are provided for user to choose. With local clustering coefficients, the feature dimension can be significantly reduced from *N* × *N* (or *K* × *K* for dHOFC) to *N* (or *K* for dHOFC). Of note, for certain network construction methods (e.g., dHOFC and SSGSR), we fixed the feature extraction methods to keep consistent with the previous studies (Chen et al., 2016; Zhang, Zhang, et al., 2019). There are many other network properties, such as the shortest path length and betweenness centrality; they could also be jointly used as network features for better capturing the network topology in the future version (Liu et al., 2018).

As for feature selection or reduction, users can choose one from two‐sample *t*‐test (*p* < .05) (Yu et al., 2016), LASSO (least absolute shrinkage and selection operator) (the feature sparsity is controlled by a hyper‐parameter *λ* that is fixed [0.05] in this version but users are allowed to change it if the number of selected features is too small or too many) (Tibshirani, 1996), and both (Zhang, Zhang, et al., 2019). This is an important step to reduce feature dimension and improve model generalizability. For certain methods, we fixed the feature extraction and selection to keep consistent with previous studies.

SVM is adopted as the classifier in BrainNetClass (Cortes & Vapnik, 1995; Misaki, Kim, Bandettini, & Kriegeskorte, 2010), which is a popular classification model and has been proved to be powerful and robust even with small sample size. We use LIBSVM v3.23 (Chang & Lin, 2011) to train SVM. We used a linear kernel and the hyper‐parameter *C* in SVM is set to 1, but users are allowed to change it if necessary (or users are advised to save the constructed networks and use other classification toolboxes that include automatic parameter tuning for classifiers, such as GraphVar).

Cross‐validation is important for classification performance evaluation but it could be done incorrectly by inexperienced users. Among many other strategies (Varoquaux et al., 2017), we provide two classic and popular cross‐validation strategies: leave‐one‐out cross‐validation (LOOCV, suitable for small sample size) and 10‐fold cross validation (suitable for large sample size). In LOOCV, the training and testing procedures are repeated for *M* times, each time leaving out a different subject for testing, and the performance is computed across the M classification results. It is similar for 10‐fold cross validation but each time one tenth of the subjects is left out for testing. The 10‐fold cross validation is repeated for 10 times (default) or more, each time with random subject partitioning. The classification performance is then averaged across all folds and all repetitions. Feature selection is conducted on the training set and the selected feature indices are applied to the testing set. If there is freely estimable parameter(s) for the parameter requested network construction methods, nested cross‐validation will be implemented for parameter optimization (see (Chen et al., 2016) for detailed procedures). Put it simple, the parameter optimization is carried out in the inner cross validation to make sure the test set in the outer cross validation is not involved in choosing parameter(s).

Classification performance is evaluated based on a battery of assessment metrics, including classification accuracy (ACC), the area under ROC curve (AUC), sensitivity (SEN), specificity (SPE), precision, balance accuracy (BAC), Youden Index (Yonden), and F‐score (Sokolova, Japkowicz, & Szpakowicz, 2006) (see (Yu et al., 2017) for the definitions). The ROC curve describes the diagnostic ability of a binary classifier when its discrimination threshold is varying. The AUC measures the probability that a classifier assigns a higher score to a randomly chosen positive example than that to a randomly chosen negative example.

### 2.3 Result display and interpretation

#### 2.3.1 Contributing features

In addition to the numeric classification performance evaluations and the ROC curve plot, users usually want to know which features contribute more (a.k.a., contributing features) or are more important to the classification. In BrainNetClass, we provide two types of feature importance measurements for the users to determine contributing features. First, the average weight derived from the SVM for each feature across all cross‐validation runs can be used as feature importance measurement. The larger the absolute weight a feature has, the more important this feature could be. Therefore, the feature importance can be represented by an *N* × *N* matrix if the features are connection coefficients and by a length‐*N* vector if the features are local clustering coefficients. Users may visualize the most important links or ROIs that may help to discover potential disease biomarkers. For dHOFC, a total of *K* matrices (each has a size of *N* × *N*) will be generated to identify the important “high‐order” nodes (i.e., a cluster of synchronized dynamic FC links), as shown in (Liu et al., 2018).

Another quantitative measurement of feature importance is the occurrence of each feature being selected in the feature selection across all cross‐validation runs (Chen, Zhang, Lee, et al., 2017; Yu et al., 2017; Zhang, Zhang, et al., 2019). The contributing features can be those consistently selected in most, if not all, of the cross‐validation runs. Similarly, it takes a form of an *N* × *N* matrix if features are connection coefficients and a length‐*N* (or length‐*K* for dHOFC) vector if features are local clustering coefficients. The more frequently a feature has been selected, the more important this feature could be. Of note, none of the previous toolboxes provides such a comprehensive contributing feature report.

#### 2.3.2 Constructed networks

Aside from the discriminative features, the pattern of the constructed brain functional networks is also informative to network neuroscience researchers. The toolbox also provides the group‐averaged brain network for each group in a form of weighted adjacency matrix. If choosing a brain network construction method that requires parameter optimization, the group‐averaged brain network constructed using the optimal parameter(s) will be generated (Chen, Zhang, Lee, et al., 2017)). If users want to save the constructed networks for all subjects separately, they are referred to use command line mode (see Section 3.3).

#### 2.3.3 Log file

Meanwhile, a full log of model configuration and performance are summarized in a log file, including the network construction method used, feature extraction and selection methods used, the ranges of parameters to optimize from, and the model evaluation method used, the suggested parameters (according to the parameter sensitivity test, see Section 2.3.4), the parameter selection occurrence (Chen, Zhang, Lee, et al., 2017), and all the numeric model performance evaluation metrics. Many of them are uniquely provided by this toolbox.

#### 2.3.4 Parameter sensitivity test and the suggested parameters

Most of the brain network construction methods require parameter tuning. It is necessary to know which parameters could be the best for the future classification to a new subject. On the other hand, one may want to test if the achieved classification performance is sensitive to specific parameter choices (if so, parameter choosing will be done more carefully in the future application). To this end, our toolbox implements a comprehensive assessment of the variations in model performance in terms of different parameters used, as did previously (Yu et al., 2017; Zhang, Zhang, et al., 2019).

Specifically, the classification model is re‐trained with each value (or each combination) of the freely estimable parameter(s) with LOOCV, which will create a bar plot showing the effects of varying parameter values on the classification accuracy. If a classification model is sensitive to the parameters, there should be a bar significantly higher than others. In this case, the user is advised to narrow down the parameter range by centering the candidate parameters on those corresponding to the peak bar. On the other hand, if the contour spanned by the bars is smooth and the peak performance is quite similar to the performance generated by the rigorous nested cross‐validation, the classification model is less sensitive to the parameters. The parameter(s) associated with the peak bar is (are) the *Suggested Parameters* for future use.

#### 2.3.5 Model robustness test and the most consistently chosen parameters

For classification studies, model robustness evaluation is equally important. Therefore, how many times a specific parameter (or a combination of parameters) is selected as the optimal parameter(s) is recorded by the toolbox and reported as *Parameter Selection Occurrence*. If the parameter was selected more frequently than others, the classification model is robust. An evenly distributed parameter selection occurrence may indicate that the model could be drastically changed in the cross‐validation (i.e., less robust or more data‐dependent). Of note, it is not necessary that the parameter(s) with the highest occurrence is the same as the suggested parameter(s), but we have observed that a good classification model resulted in the suggested parameter(s) same as that with the highest occurrence (see examples in Section 4).

## 3 TOOLBOX DESIGN AND USAGE

### 3.1 Functional modules and designing logics

BrainNetClass (v1.0) consists of several sequentially executed modules (Figure 1). The toolbox takes region‐averaged rs‐fMRI time series as inputs, which can be generated by other toolboxes, such as SPM^3^ or FSL^4^. The user does not need to do any further preprocessing. However, the user needs to decide which brain network construction method to use beforehand. All the available network construction algorithms are organized into *Type I* (those without any parameter to optimize) and *Type II* (those with parameter optimization required). The parameter‐required network construction methods gain certain advantages but need more work to test the model robustness and parameter sensitivity. How to choose the proper network modeling algorithm will be provided in Section 5.1. For some network construction methods, default feature extraction and feature selection are provided and a default parameter range will also be provided to facilitate decision making. However, the user can change the default parameter range (see an example in Section 2.3.4). The choice of cross‐validation method depends on sample size.

Before starting, the required memory will be estimated and displayed to users. The memory required and the computing time are proportional to the number of ROIs and the total sample size and depend on the network construction method. Generally, dHOFC consumes more memory, and SR‐related methods with two estimable parameters need longer computational time. When the process has successfully finished, the toolbox generates multiple results in the result folder (users will have freedom to choose any network visualization software to visualize the contributing features) and the classification performance is shown in the GUI. The figures and the log file summarizing major methods and results are generated to assist paper writing. It is advocated that not only the diagnosis accuracy but also the constructed networks, contributing features, and model robustness assessment should be reported in the paper. All the model information can be retrieved afterwards. The user is advised to interpret the results with their domain knowledge.

### 3.2 Step‐by‐step usage

**Figure 2:**
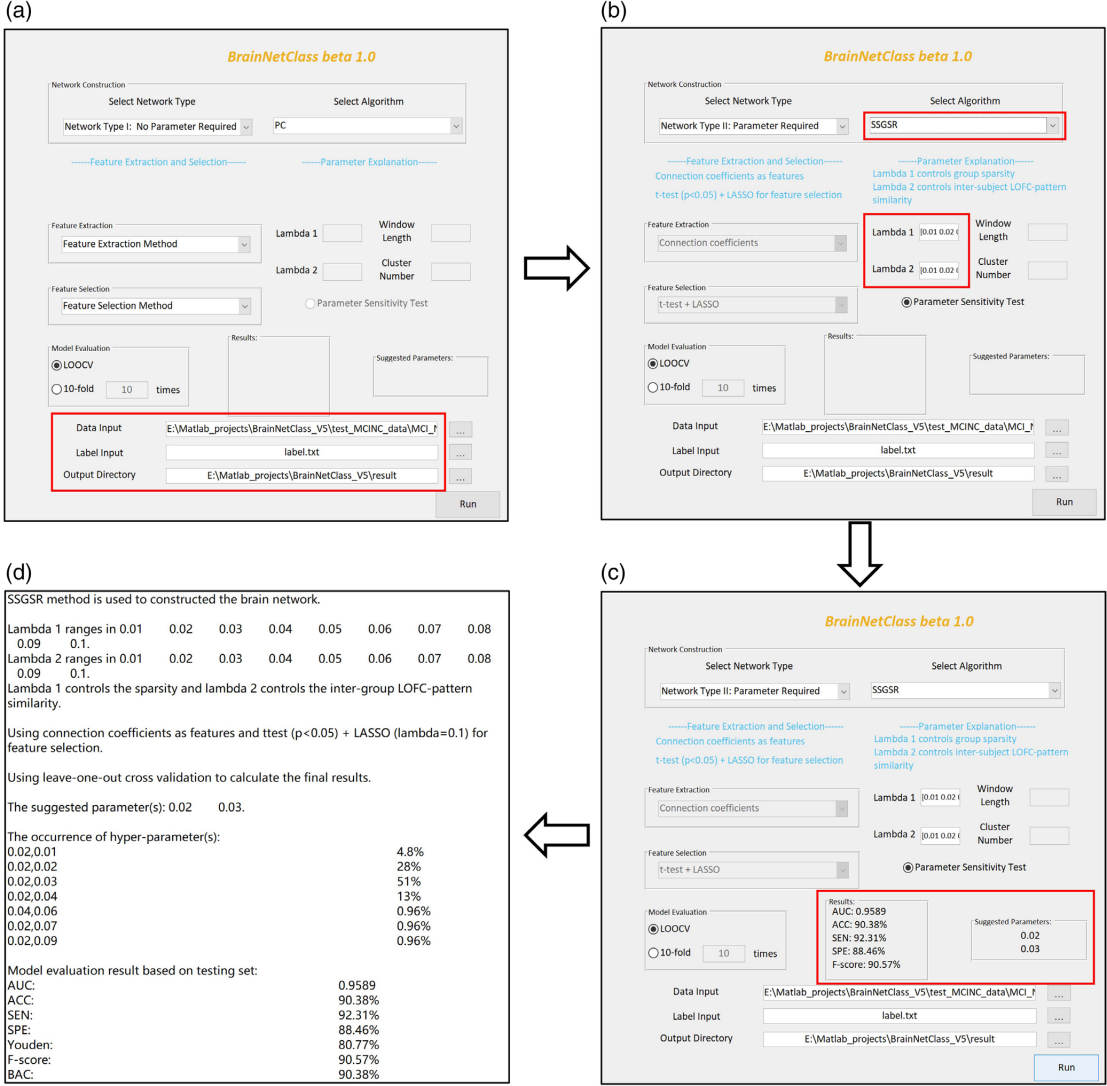
Step‐by‐step setup in BrainNetClass and its result report, including (a) Specifying inputs and output, (b) selecting network construction method as well as feature extraction/selection and validation methods, (c) model evaluation, and (d) log file generation

We give a brief walkthrough of the toolbox (see more details in the manual^5^):

1. Specify region‐averaged rs‐fMRI time series data of all subjects and a text‐formatted label file containing a column of the labels for all subjects (e.g., 1 for patient and −1 for control) in the same order. Specify output directory (Figure 2a).
2. Choose network construction method. The user may need to specify the parameter range(s) or use the default setting. See brief explanations of the parameter meanings (Figure 2b).
3. Select or use predefined feature extraction and feature selection methods. See brief explanations of the methods selected.
4. Choose a cross‐validation method. If choosing 10‐fold cross‐validation, specify how many times the cross‐validation will be repeated.
5. All set. Clicking the Run button and wait for all processes completed, as indicated by an “*All Jobs Completed*” window popping out. Some important results will be printed on the result panel and the suggested parameters panel (Figure 2c).
6. A full log for a hassle‐free report is generated in the result folder (Figure 2d).
7. The users may repeat the steps 1–6 to generate results from other baseline methods, such as PC and SR, for comparison purpose.

### 3.3 Useful commands

Our toolbox also provides helpful and efficient commands that can be executed to conduct entire works for skilled users and they can be run easily with minimum coding on computing clusters or servers without using the GUI. See complete list of the commands or functions in the manual.

We provide one function that can save the constructed brain network for any user who wants to perform further analysis based on the saved networks constructed by any of the network construction methods mentioned above. Also, some users may want to choose other methods or algorithms for feature selection and classification and they can perform further analysis using other machine learning based toolboxes (e.g., WEKA (Hall et al., 2009)) with more advanced machine learning based algorithms (such as optimizing the SVM hyper‐parameters). We can take the brain network constructed by user themselves using other methods as input, and perform feature extraction, feature selection, classification and detect discriminative features.

## 4 TOOLBOX VALIDATIONS

To further evaluate the effectiveness of our toolbox, we applied it to real rs‐fMRI datasets. For the first three applications, we chose one advanced method to construct the brain network and compared it with two baseline methods (PC and SR). In the fourth application, we chose a multi‐center dataset and tested the cross‐center generalization ability. All the experiments were conducted using Matlab version 2018a based on a Window desktop computer with six CPU kernels and 64Gb physical memory.

### 4.1 Application 1: SSGSR‐based MCI diagnosis

#### 4.1.1 Materials and methods

The dataset is from the Alzheimer's Disease Neuroimaging Initiative (ADNI) database^6^. MCI is a transitional stage between Alzheimer's Disease (AD) and cognitively normal subjects, which has a high chance to progress to AD (Gauthier et al., 2006). This application is to demonstrate the feasibility of timely detection of MCI based on brain functional networks.

The rs‐fMRI data from 52 MCI patients and 52 matched normal controls (NCs) were selected from the ADNI‐2 database. The two groups were all scanned using 3.0‐T Philips scanners with the same imaging protocols^7^. The data are from multiple imaging centers but imaging quality control was carefully carried out to make sure the across‐site consistency. The rs‐fMRI data were preprocessed by using SPM8 with a standard procedure described elsewhere (Chen, Zhang, Zhang, et al., 2017). Automated Anatomical Labeling (AAL) template was used to extract ROI‐averaged time series from the 116 ROIs.

SSGSR can reduce individual variability by using the group sparsity constraint and improve between‐group separability. Since the MCI group may have large individual variability, we applied SSGSR to construct brain networks for all the subjects. The parameters including *λ*_1_ (controlling the group sparsity) ranging in [0.01, 0.02, …, 0.1] and *λ*_2_ (controlling inter‐subject network similarity) ranging in [0.01, 0.02, …, 0.1]. The connection coefficients were used as features. Two‐sample *t*‐test (*p* < .05, uncorrected) was adopted to initially remove less discriminative features and LASSO was further applied to further select features. The MCI versus NC performance was compared with those of PC and SR. LOOCV was adopted to evaluate the performance. The optimal *λ*_1_ and *λ*_2_ were determined with inner LOOCV.

#### 4.1.2 Results

**Figure 3:**
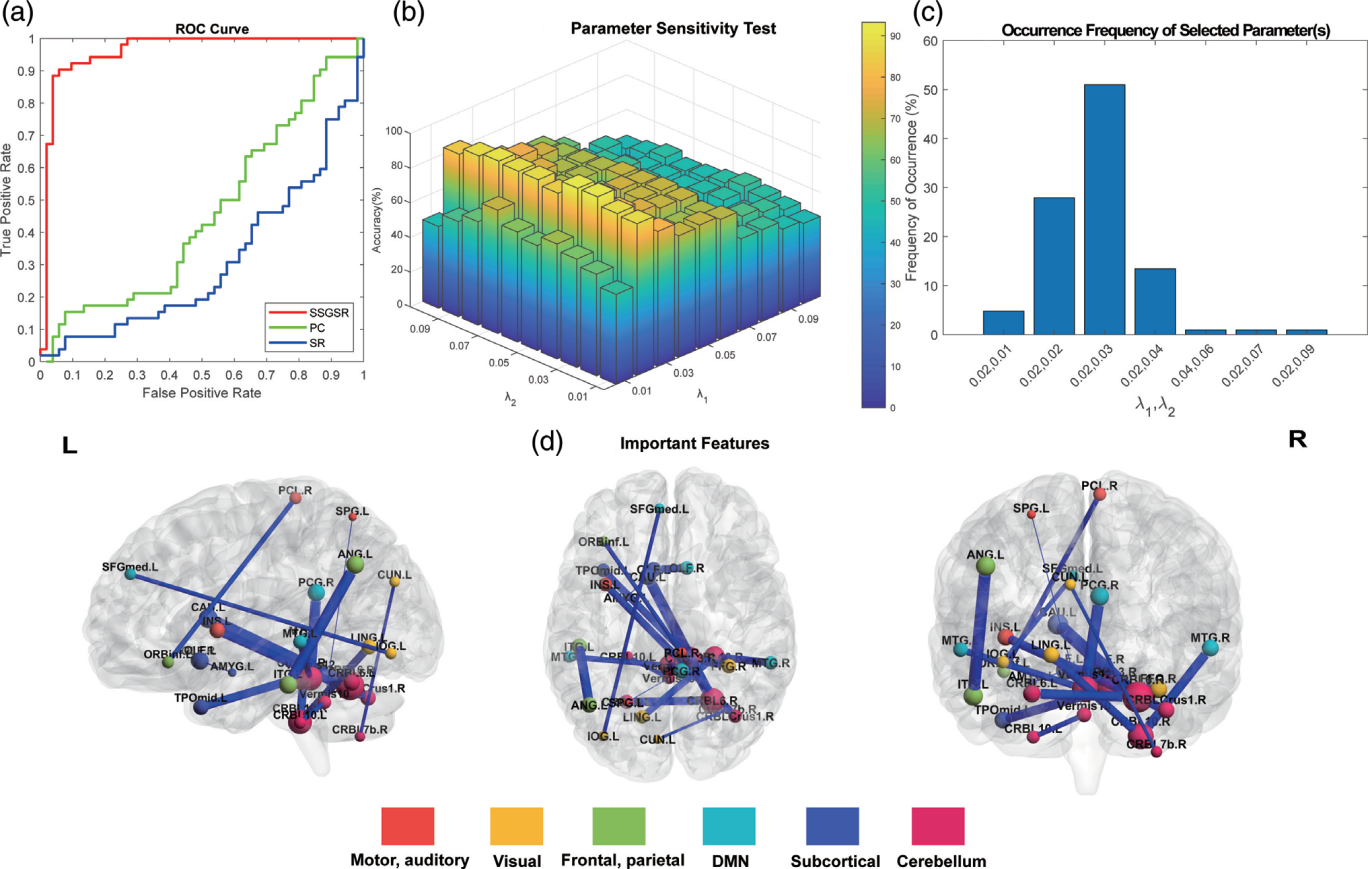
MCI diagnosis results, including ROC curve comparisons between SSGSR and PC/SR (a), parameter sensitivity testing result (b), model robustness evaluation (c), and the contributing features suggested by the SSGSR‐based classification according to the feature selection occurrence (d). The contributing FC links were visualized by using BrainNet Viewer, with only the features being 100% selected shown, where the edge thickness represents the averaged absolute weight and the node size represents the averaged absolute weights associated with each node. FC, functional connectivity; SSGSR, strength and similarity guided GSR

SSGSR achieved much better performance than that of PC and SR (Table 2, Figure 3a). As shown in Figure 3b, the model was more sensitive to *λ*_1_ than *λ*_2_. The suggested parameters are *λ*_1_ = 0.02 and *λ*_2_ = 0.03. The classification accuracy is 90.38% yielded with nested LOOCV (Table 2), close to the accuracy obtained by the suggested parameters (93.27%, Figure 3b). The most frequently selected parameters are the same as the suggested parameters, as shown in Figure 3c.

**Table 2:** MCI diagnostic performance by using SSGSR, PC, and SR

	AUC	ACC	SPE	Youden	BAC	SEN	F‐score	Time
SSGSR	0.9589	90.38%	88.46%	80.77%	90.39%	92.31%	90.57%	4.63 hr
PC	0.5680	51.93%	50.00%	3.85%	51.93%	53.85%	52.83%	12.27 s
SR	0.3081	34.62%	38.46%	−30.77%	34.62%	30.77%	32.00%	0.66 hr

Abbreviations: ACC, accuracy; AUC, area under ROC curve; BAC, balance accuracy; dHOFC, dynamics‐based HOFC; PC, Pearson's correlation; SEN, sensitivity; SPE, specificity; SR, sparse representation; SSGSR, strength and similarity guided GSR.

The prognostic connectivity features that were 100% selected during the LOOCV were plotted in Figure 3d, with link thickness and node size indicating the feature importance in terms of MCI classification. A total of 17 discriminative connections were identified. Most of them are closely related to AD pathology as suggested by previous studies (Buckner et al., 2005; Buckner, Andrews‐Hanna, & Schacter, 2008; Frisoni, Prestia, Rasser, Bonetti, & Thompson, 2009; Jacobs et al., 2012; Li et al., 2012; Thomann et al., 2008; Wee et al., 2016), such as the default mode network and the connections between cerebellum and cortical regions. All the results shown here were generated by our toolbox.

### 4.2 Application 2: SGR‐based brain‐state classification

#### 4.2.1 Materials and methods

Eyes open (EO) and eyes closed (EC) states have been used in many rs‐fMRI studies, and several studies have shown that there are fundamental differences between these two states (Liang et al., 2014; Yu‐Feng et al., 2007; Zhou, Wang, Zang, & Pan, 2018). This dataset was also provided as” Toydata1.zip” (see” Toydata2.zip” for a simplified version for a quick run) in a text format as well as” EC.mat” and” EO.mat” in the BatchExamples folder in https://github.com/zzstefan/BrainNetClass. In this experiment, we aimed to evaluate the feasibility of individualized brain‐state classification (EO vs. EC) based on brain FC networks. We also aimed to identify important FC links that played significant roles in the classification.

The data from 48 (22 females) college students (aged 19–31 years) was downloaded from a publicly available dataset, Beijing Eyes Open Eyes Closed Study^8^. The rs‐fMRI data during EC and EO were separately acquired from the same subject using a SIEMENS TRIO 3.0‐T scanner at the Beijing Normal University and the imaging protocol can be found in (Liu, Dong, Zuo, Wang, & Zang, 2013). One subject was excluded due to the incomplete data. The conventional rs‐fMRI preprocessing was conducted using DPABI (Yan et al., 2016). None of the subjects was excluded due to excessive head motion (>2 mm in displacement or >2^°^ in rotation, or with mean framewise displacement [FD] > 0.5 mm).

SGR was used to construct brain networks consisting of 116 ROIs according to the AAL template. The parameters of the SGR model were set up as follows: *λ*_1_ = [0.01, 0.02, …, 0.1] and *λ*_2_ = [0.01, 0.02, …, 0.1]. All the other settings are the same as those in Application 1. The performance of the SGR model was compared with those of PC and SR based on LOOCV.

#### 4.2.2 Results

**Figure 4:**
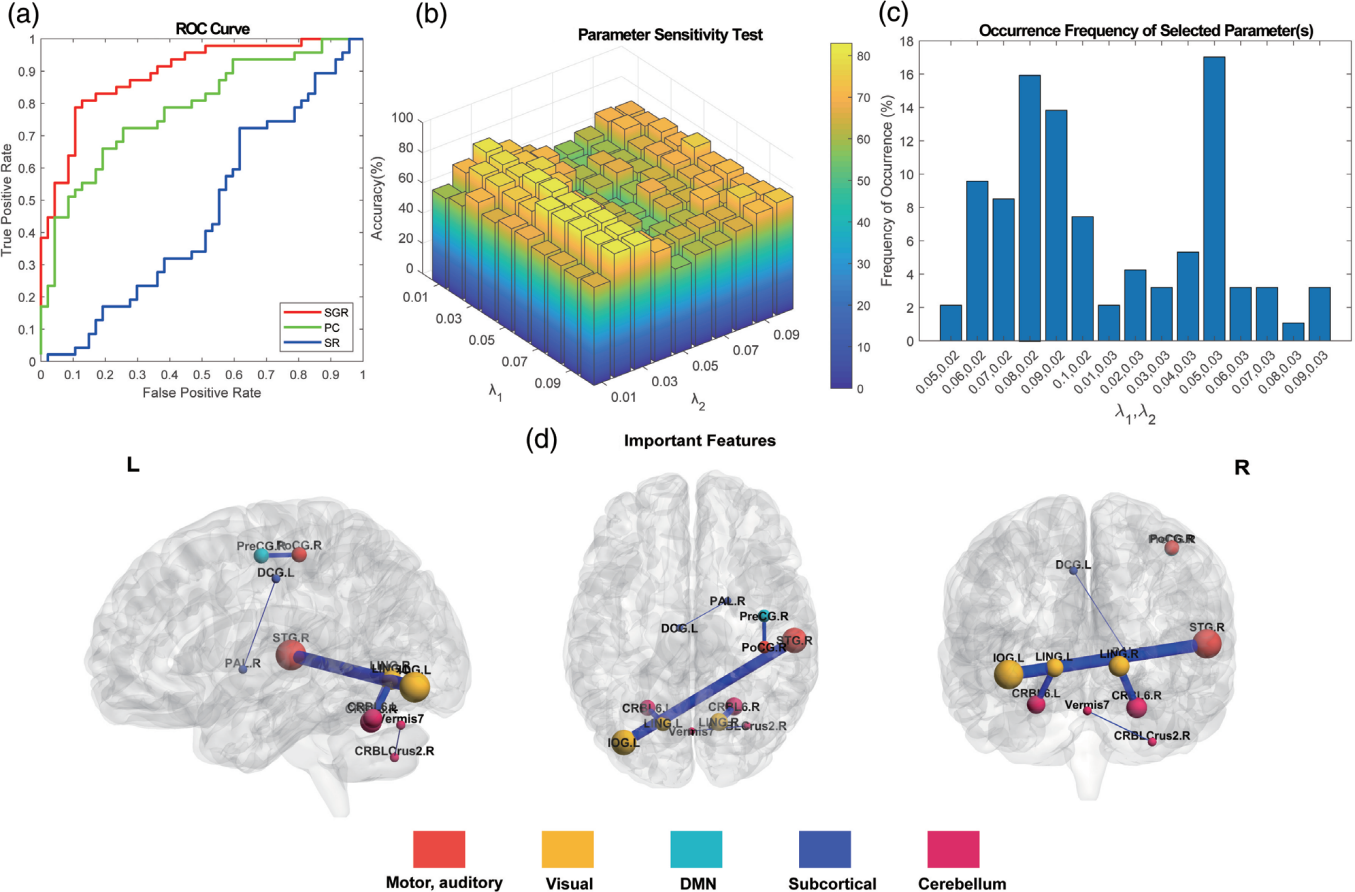
Eyes closed versus eyes open (EC vs. EO) classification results, including ROC curves for the SGR, PC, and SR (a), result from parameter sensitivity test (b), parameter selection occurrence (c), and the important features identified (d). PC, Pearson's correlation; SGR, sparse group representation; SR, sparse representation

The performance of SGR was better than that of PC and SR, though we found a method as simple as PC can result in a satisfactory accuracy as well (Figure 4a, Table 3). Parameter sensitivity test shows that the results are not very sensitive to different parameters (Figure 4b), and the suggested parameters (*λ*_1_ = 0.08 and *λ*_2_ = 0.02) resulted in a similar accuracy as that derived from nested LOOCV (82.98% vs. 79.79%). The suggested parameters are the second most selected parameters (Figure 4c). The top two mostly selected parameters were only selected for 16 and 15 times (~17% and 16% of all the LOOCV runs), indicating that the model robustness requires future check. In this case, the PC could be the most suitable model, as it involves no parameter tuning. Nevertheless, we plotted six most consistently selected connections in Figure 4d. Most of them are consistent with the previous studies, such as the FC links in the sensorimotor and visual networks (Zou et al., 2015; Zhou, Wang, et al., 2018).

**Table 3:** The classification performance of EC/EO by SGR, PC, and SR

	AUC	ACC	SPE	Youden	BAC	SEN	F‐score	Time
SGR	0.8927	79.79%	76.60%	59.98%	79.79%	82.98%	80.41%	4.81 hr
PC	0.7841	71.28%	70.21%	42.55%	71.28%	72.34%	71.58%	9.53 s
SR	0.4595	43.62%	44.68%	−12.77%	43.62%	42.55%	43.01%	1.31 hr

Abbreviations: ACC, accuracy; AUC, area under ROC curve; BAC, balance accuracy; dHOFC, dynamics‐based HOFC; PC, Pearson's correlation; SEN, sensitivity; SGR, sparse group representation; SPE, specificity; SR, sparse representation.

### 4.3 Application 3: dHOFC‐based depression diagnosis

#### 4.3.1 Materials and methods

Accurate diagnosis of treatment‐naïve and first episode major depressive disorder (MDD) can be quite challenging (Souery et al., 1999). Computer‐aided diagnosis is necessary as it can not only conduct objective diagnosis but also can identify depression‐related pathological changes in brain. Therefore, we performed a brain network‐based individualized classification between MDD and NC using our toolbox.

The major depressive disorder patients, compared to the NCs, may have subtle changes in terms of the static FC networks. As we stated in the algorithm selection recommendation in Discussion, dynamic FC‐based dHOFC could better capture the differences between these two groups. The data was from the First Affiliated Hospital, Guangzhou University of Chinese Medicine, consisting of 53 MDDs and 53 matched NCs (age between 19–32 years). They were all acquired using a 3.0‐T GE Signa HDxt scanner. The data was also used in Zheng et al. (2019) with similar method but without parameter optimization. To demonstrate the effectiveness of our toolbox, we re‐applied dHOFC to the same data but allowing the parameters to be optimized based on a nested LOOCV. We built dHOFC networks for the diagnosis because the dynamic FC‐derived dHOFC has been found to be quite sensitive in brain disease diagnosis (Chen et al., 2016; Zhang, Zhang, et al., 2017). The AAL template was used and the window size was optimized from [50, 60, …, 120] (step size was set to 2). The number of clusters was optimized from [100, 200, …, 800]. Local clustering coefficients were used as features and LASSO was used for feature selection (Chen, Zhang, Zhang, et al., 2017). We compared the performance of dHOFC with that of PC or SR. Due to the limited sample size, LOOCV was used to evaluate performance.

#### 4.3.2 Results

**Figure 5:**
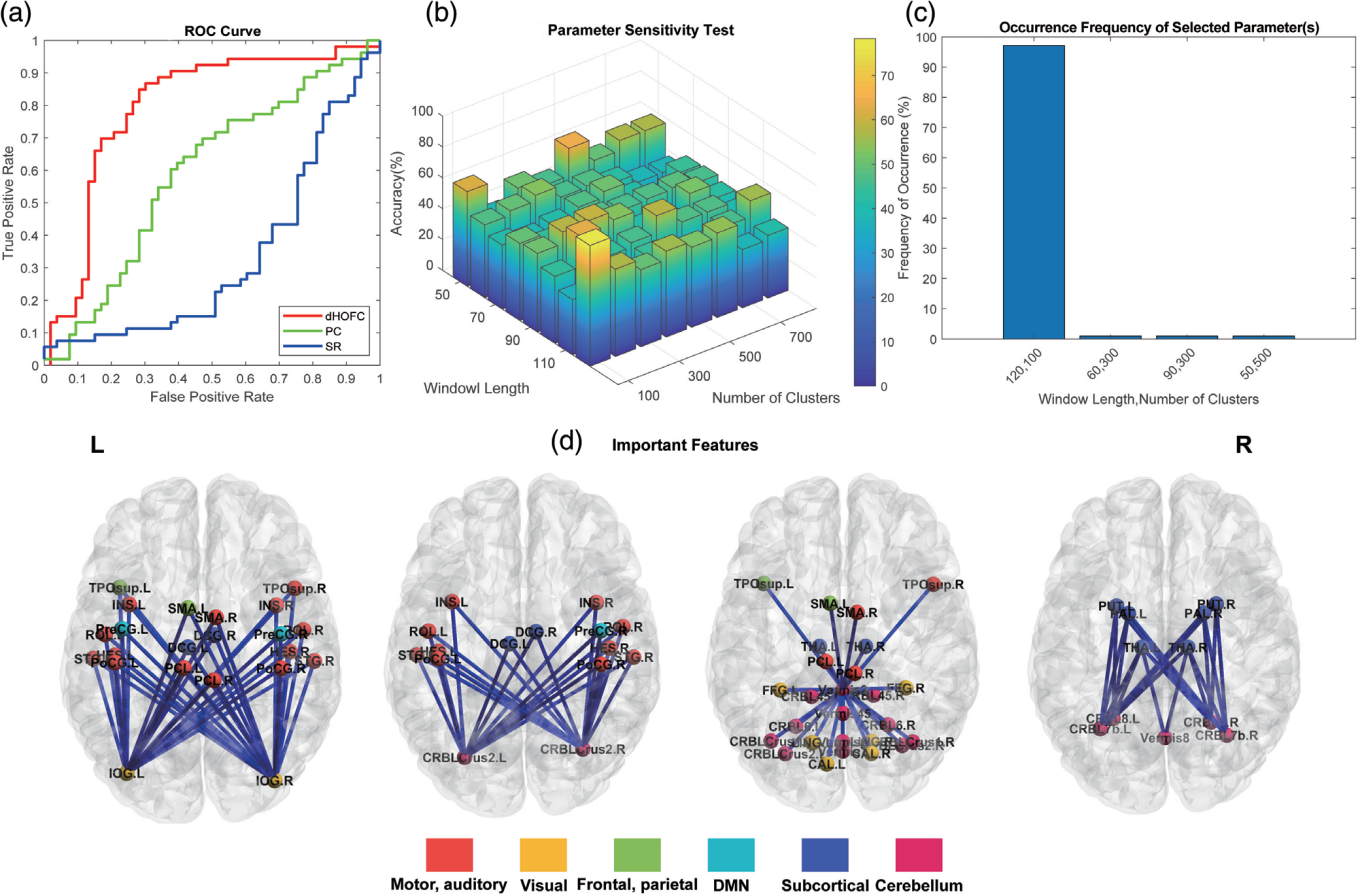
MDD versus NC classification results based on dHOFC, including the ROC curves of dHOFC, PC, and SR (a), parameter sensitivity testing result (b), the model robustness testing result (c), and the top four contributing features (d). MDD, major depressive disorder

As shown in Table 4 and Figure 5a, dHOFC outperformed PC and SR. The MDD diagnosis accuracy (76.42%) based on dHOFC was close to the upper limit of the accuracies in the parameter sensitivity test (78.30%, Figure 5b). The suggested window length was 120 and the suggested number of clusters was 100. Figure 5c shows that the most frequently selected parameters are the same as the suggested parameters. Importantly, the model seemed quite robust, as there was a dominant parameter combination being selected (in ~95% of the total LOOCV runs).

**Table 4:** The classification performance of MDD/NC by dHOFC, PC, and SR

	AUC	ACC	SPE	Youden	BAC	SEN	F‐score	Time
dHOFC	0.7964	76.42%	73.58%	52.38%	76.42%	79.25%	77.06%	2.53 hr
PC	0.5931	60.38%	49.06%	20.76%	60.38%	71.70%	64.41%	13.83 s
SR	0.3300	33.96%	39.62%	−32.08%	33.96%	28.30%	30.00%	0.73 hr

Abbreviations: ACC, accuracy; AUC, area under ROC curve; BAC, balance accuracy; dHOFC, dynamics‐based HOFC; MDD, major depressive disorder; PC, Pearson's correlation; SEN, sensitivity; SPE, specificity; SR, sparse representation.

Each feature in the dHOFC network are not simple pairwise FC links between two regions. Instead, each dHOFC feature constituted several FC links with temporally synchronized FC dynamics. We visualized four features that were consistently selected, each of which was shown as a group of connections (Figure 5d). The connections between the visual and sensorimotor networks, as well as the connections between the subcortical and cerebellar networks were indicated to be contributive to the MDD classification, which are consistent with the previous studies (Kaiser, Andrews‐Hanna, Wager, & Pizzagalli, 2015; Mulders, van Eijndhoven, Schene, Beckmann, & Tendolkar, 2015; Zeng et al., 2012; Zheng et al., 2019).

### 4.4 Application 4: dHOFC‐based autism spectrum disorder diagnosis

#### 4.4.1 Materials and methods

Autism spectrum disorder (ASD) is characterized by impairments in social cognition, communication with narrow and repetitive behaviors and interests (Lord, Cook, Leventhal, & Amaral, 2000). We adopted the data from the Autism Imaging Data Exchange (ABIDE I) dataset and performed an independent‐center cross validation (i.e., leave‐center‐out cross validation) to evaluate the generalization ability of our network construction method from one center to another. ABIDE is a consortium that provides collected rs‐fMRI ASD and matched controls data with the purpose of sharing data in the scientific community (Di Martino et al., 2014; Zhao, Zhang, Rekik, An, & Shen, 2018). Processed rs‐fMRI data was downloaded from the Preprocessed Connectomes Projects^9^. Data were selected from the DPARSF pipeline^10^ with the details listed elsewhere. The time series was extracted using the AAL template. We selected 6 sites: (1) California Institute of Technology (Caltech, 19/18 ASD/controls), (2) Kennedy Krieger Institute (KKI, 10/25 ASD/controls), (3) Social Brain Lab, Netherlands Institute for Neuroscience (SBL, 14/12 ASD/controls), (4) San Diego State University (SDSU, 12/21 ASD/controls), (5) Stanford University (Stanford, 17/19 ASD/controls), and (6) Trinity Centre for Health Sciences (Trinity, 21/23 ASD/controls). We left each of the six centers' data out as a testing set (e.g., KKI) and used the data from remained five sites (e.g., Caltech, SBL, SDSU, Stanford, and Trinity) as a training set. We also built the dHOFC networks because of its established sensitivity in brain disease diagnosis. We used 10‐fold cross validation in the inner cross‐validation with the training data to optimize the estimable parameters, window size and the number of clusters. The parameter ranges were set to be the same as those in Section 4.3.1. We implemented the same performance evaluation methods to evaluate performance and generalization ability.

#### 4.4.2 Results

The dHOFC's performance in separating ASD subjects from the controls ranged from 53.33% to 74.29% (61.04% ± 7.86%), according to the leave‐center‐out cross validation. The suggested window length was 90 and the suggested number of clusters for dHOFC network construction was 400, based on the best performance. Our method achieved generally acceptable performance even with a stringent cross‐center‐validation.

## 5 DISCUSSION

In this article, we presented an easy‐to‐use Matlab toolbox, BrainNetClass v1.0, for brain network construction and network‐based classification. The toolbox integrates state‐of‐the‐art network construction methods and provides a comprehensive and rigorous solution of individualized rs‐fMRI‐based classification. It was designed to encourage clinical applications using brain functional networks derived from rs‐fMRI. The target users are the clinicians with data and domain knowledge but are less familiar with machine learning. The toolbox provides a standard and widely‐adopted pipeline to minimize possible confusion with quite a lot freedom such as parameter optimization. It generates multi‐facet qualitative (e.g., network visualization) and quantitative (e.g., model performance and robustness) results and allows users to explore the contributing features to further push the boundary of imaging‐based machine learning studies in the clinical field. Effectiveness of the toolbox was proven by real rs‐fMRI experiments with varied classification goals. The paper will be of broad interests to clinicians and neuroscientists. BrainNetClass is the first toolbox that focused on advanced network construction and network‐based classification result interpretation.

### 5.1 Algorithm selection determination

Our toolbox extends the conventional network construction (PC‐based FC network) to high‐order FC networks and SR‐based networks. It brings a new requirement to choose proper network construction methods as previous toolboxes did not provide such an option. While we separate these network construction algorithms into two categories in the toolbox based on the requirement of parameter optimization, we could also separate them into pairwise (PC, tHOFC, aHOFC, and dHOFC) and multi‐region‐based methods (all the SR‐based algorithms). While the test–retest reliability of the PC and all the HOFC algorithms has been verified (Zhang, Chen, et al., 2017), a similar study has not been done for the SR‐based network construction. Therefore, if users want to use proved reliable network construction methods, PC, tHOFC, and aHOFC are suggested. Compared to PC, tHOFC and aHOFC are more robust to noise and, most importantly, they could provide supplementary information to PC (Zhang, Chen, et al., 2016; Zhang, Giannakopoulos, et al., 2019). From another viewpoint, only dHOFC utilizes dynamic FC, while all other methods still focus on static FC. These general considerations should be taken when choosing network construction algorithms.

Network construction method could be chosen based on specific research question. For example, it has been shown that there are fewer differences in the PC‐based static FC networks comparing patients with mental disorders with NCs (Zheng et al., 2019). Instead, dynamic FC could reveal more subtle differences associated with mental disorders (Demirtaş et al., 2016; Kaiser et al., 2016; Rashid, Damaraju, Pearlson, & Calhoun, 2014). By using dynamic FC, dHOFC could capture higher level of complex interactions among brain regions and might perform better than the conventional low‐order static FC approaches (Zheng et al., 2019). Since most biological networks are intrinsically sparse (Rubinov & Sporns, 2010), SR has been widely used in biological signal analysis, such as electroencephalography (EEG) (Wen, Jia, Lian, Zhou, & Lu, 2016; Zhang, Zhou, et al., 2016; Zhang et al., 2018) and rs‐fMRI studies (Lv et al., 2015; Suk, Wee, Lee, & Shen, 2015). Another advantage of the SR‐based brain network construction is that weak FCs that might be highly affected by noise and artifacts can be suppressed without an arbitrarily defined threshold (Wee et al., 2014). Therefore, for data with potentially higher noise level (e.g., the temporal signal‐to‐noise ratio is smaller than 50 (Murphy, Bodurka, & Bandettini, 2007), or the subject's head motion is relatively large with mean frame‐wise displacement larger than 0.25 mm (Shen et al., 2016)), SR‐based methods can be used to suppress the excessive noise. Of note, the parameter(s) associated with this type of methods should be tuned rather than preset to achieve an adaptive sparsity adjustment to specific datasets. Users are recommended to assess model robustness and parameter sensitivity according to Sections 2.3.4 and 2.3.5. Users are also advised to check Section 2.1.2 for specific benefits that each method can offer. For example, the WSR, WSGR, and SLR methods can make the estimated network more biologically meaningful (Qiao et al., 2016; Yu et al., 2017). If certain subjects have relatively higher level of noise and artifacts compared to others, the PC‐based networks could show excessive individual variability that could overwhelm a true group effect. The group‐wise sparse representation, such as GSR or SSGSR, can make the networks topologically more identical across subjects and facilitate classification (Zhang, Zhang, et al., 2019).

We advise the user to include the results from PC and SR as baseline methods (see Section 3.2), as these two methods are traditionally used to estimate full correlation and partial correlation, respectively. Their results can be served as baseline to compare with that from a more advanced network construction method. By comparing their results, users can have a better understanding of the advanced network construction methods and the benefit they could offer.

### 5.2 Comparison with other similar toolboxes

There exist several freely available packages for machine learning study with neuroimaging data, including PyMVPA, Sci‐kit Learn, PRoNTo, and GraphVar. We briefly compare them with our toolbox, as summarized in Table 5. PyMVPA (Hanke et al., 2009) and Scikit Learn (Abraham et al., 2014) are two sophisticated and flexible software packages primarily written in Python. The wide applications of these two packages allow them to be easily combined with other neuroimaging and machine learning packages. See many applications to magnetoencephalography (MEG), EEG, structural MRI, and fMRI (Abraham et al., 2014; Guntupalli, Feilong, & Haxby, 2018; Hanke et al., 2009). However, these two packages only provide command line‐based analysis without any GUI, which is not easy for less experienced users to use. PRoNTo and GraphVar are both Matlab toolboxes with GUI and provide abundant functions, including pattern recognition analysis of neuroimaging data. PRoNTo aims at providing a comprehensive and user‐friendly framework for multivariate analysis of neuroimaging data (Schrouff et al., 2013). GraphVar provides pipelined machine learning‐based model construction, validation, and exploration, which can use various graph attributes to model their relationship with other variables, thus is quite flexible in different neuroimaging applications (Kruschwitz et al., 2015; Waller et al., 2018).

**Table 5:** Comparison of the main features of the available software packages

	PyMVPA	Scikit‐learn	PRoNTo	GraphVar	BrainNetClass
Inputs	Numpy arrays, *.txt, NIFTI, EEP	Numpy arrays, metadata, *.txt, *.csv	MRI/fMRI feature maps (NIFTI)	Time series, connectivity matrix (*.mat)	Time series (*.txt,*.csv)
Language	Python	Python	Matlab	Matlab	Matlab
Voxel/network‐wise	Voxel	Voxel/network	Voxel	Network	Network
Interface	Command line	Command line	GUI, command line	GUI	GUI, command line
Static or dynamic FC^a^	Static	Static	Static	Static, dynamic	Static, dynamic
Result display^b^	×	×	✓	✓	✓
Network construction	×	×	×	✓	✓
High‐order FC	×	×	×	×	✓
SR (and its variants)	×	×	×	×	✓
Contributing features	×	×	✓	✓	✓
Parameter sensitivity/robustness test	×	×	×	✓^c^	✓^d^

Abbreviations: FC, functional connectivity; GUI, graphical‐user‐interface; SR, sparse representation.

a Dynamic FC means whether toolbox provides any direct package to let the users calculate dynamic FC.

b Result display means displaying some of the results directly on the software panel.

c Model robustness test is performed in GraphVar via permutation test.

d For network construction methods with freely estimable parameter(s).

Compared to our toolbox, these toolboxes did not provide plenty (if any) state‐of‐the‐art network construction methods such as the SR‐based algorithms and the high‐order network construction methods. We think that the network construction is as equal as, if not more important than, classification, and that the network construction is becoming more and more important in the network neuroscience. A good network construction method can play an important role in the subsequent network‐based classification.

Compared to these toolboxes, BrainNetClass provides comprehensive functions for result display, classification model evaluation, and interpretation (Section 2.3). Many practical features that have been reported in the previous disease classification studies are included, such as saving important features for visualization and constructed networks visualization (Chen, Zhang, Lee, et al., 2017), as well as parameter sensitivity test (Yu et al., 2017; Zhang, Zhang, et al., 2019). Some features that are essential for clinical applications such as model robustness test were not even reported in the previous studies. The above mentioned features are all provided by our toolbox. For advanced users, we also provided command line mode for more flexible and efficient analysis.

### 5.3 Compatibility and computational requirement

The toolbox works well with Matlab 2016a and all later versions on a Linux desktop computer or Windows Personal Computer or computing servers. All the experiments were performed on a Windows‐based desktop computer, with 64 GB memory, i7‐8700K CPU @ 3.70 GHz. We show the computational time of each experiment in Tables 2, 3, and 4.

For a typical desktop personal computer with 2–4 cores and 8–16 GB physical memory, it can meet the computation requirement of ~100 samples with a small range of parameters to estimate. For the methods requiring no parameters, it can handle a larger sample size and will not take too long to finish the whole process. For methods requiring parameter tuning, BrainNetClass can distribute network constructions with different combinations of the parameter(s) to different CPU cores using the Matlab parallel computing modules to save computational time. We also provide 10‐fold cross validation if the study involves a larger sample size, as it will significantly decrease the computational time. The computational time and resource is proportional to the sample size. The prediction performance and its stability exponentially increased with sample size as suggested by Cui and Gong (2018). They also found that the average prediction accuracy and its stability appear to reach plateau when sample size reaches 200–300, regardless of the classification algorithm used and a minimum sample size of 200 is recommended (Cui & Gong, 2018). For studies involving such sample size, we recommend using a computer with more physical memory (e.g., at least 32 GB for ~200 samples or larger for more samples). The computational time is also related to the ROI template used to extract the rs‐fMRI time series. We compared the computational time in the EC/EO classification experiment using the Dosenbach's 160‐ROI atlas (Dosenbach et al., 2010) to extract time series. The computational time increased to about 6.68 hr compared to that with 116 ROIs (4.81 hr) and the computational complexity also increased.

### 5.4 Limitations and future works

We provide BrainNetClass to meet the urgent call for standardization of the methodology with an emphasis on (re‐evaluation of) the reproducibility, generalizability, and interpretability of the (existing) network‐based classification. It is yet in its first official version and unavoidably has limitations. First, it now only allows users to conduct two‐class classification. We will add support vector regression (SVR) and multi‐class classification in the future. Second, more feature extraction options will be provided beyond the current two type of features (FC links and local clustering coefficients) to better characterize network topology and further boost classification performance. Third, more sophisticated feature selection methods, such as ElasticNet and SVM wrapped method (recursive feature elimination (Duan, Rajapakse, Wang, & Azuaje, 2005)), might be implemented in the future. Fourth, more options of classifiers, such as random forest and Naïve Bayes, could be provided, and the final classification result may come from the ensemble of multiple classifiers to improve performance. Of note, the toolbox focuses more on brain network construction and it will include more flexible machine learning strategies in its future version. Fifth, more complex network definitions (e.g., hyper‐connectivity‐based network (Jie, Wee, Shen, & Zhang, 2016)) and more dynamic FC‐based network construction methods (e.g., the variability of the dynamic FC (Chen, Zhang, Zhang, et al., 2017)) should be added. Other effective inter‐regional interaction parametrization methods (e.g., tangent‐based parametrization of covariances (Dadi et al., 2019)) should be included. In addition, combining different networks (constructed by different methods) into ensemble learning could be promising in which the supplementary information provided by different network construction methods can be effectively fused to achieve better classification performance. Sixth, the optimization of parameters for network construction and classification could be the most important yet difficult problem in the current studies. At present, we only allow at most two freely estimable parameters at the current stage to compromise between the computational time/memory required and the model flexibility. In the future, with well‐designed parameter optimization strategies (e.g., adaptive parameter range determination), we might allow more parameters to be simultaneously optimized, such as hyper‐parameter tuning in SVM. Finally, SSGSR and GSR require all data to construct the brain network, which reduced the flexibility of both methods. This could be solved by developing a transfer learning‐based model for brain network construction for the testing subjects. The brain networks constructed by SSGSR from training sets could be used as references and as prior constraints into a subsequent sparse representation‐based model for the testing subjects.

## 6 CONCLUSIONS

We introduce a novel, Matlab GUI‐based, open‐coded, fully automated brain functional network construction and classification toolbox, namely BrainNetClass. It allows users to construct brain networks with advanced methods and conduct rigorous feature extraction/selection, parameter optimization, classification, and model evaluation in a standard and well‐accepted framework. This toolbox is friendly to neuroscientists and clinicians for facilitating their function connectomics‐based disease diagnosis or classifications with comprehensive yet intuitive and interpretable results. It is helpful for standardizing the methodology and boosting the clinical application of neuroimaging‐based machine learning with improved reproducibility, generalizability, and interpretability. The toolbox, manual, and exemplary datasets are available at https://github.com/zzstefan/BrainNetClass.

## CONFLICT OF INTEREST

The authors declare no potential conflict of interest.

7

## Data Availability

The code, toolbox manual, and testing data are available at https://github.com/zzstefan/BrainNetClass.

## Endnotes

1
http://www.fil.ion.ucl.ac.uk/spm/software/spm12/

2
https://github.com/jiayuzhou/SLEP

3
http://www.fil.ion.ucl.ac.uk/spm/software/spm12/

4
https://fsl.fmrib.ox.ac.uk/fsl/fslwiki

5
https://github.com/zzstefan/BrainNetClass/blob/master/Manual_v1.1.pdf

6
http://adni.loni.usc.edu

7
http://adni.loni.usc.edu/methods/documents/mri-protocols

8
http://fcon_1000.projects.nitrc.org/indi/retro/BeijingEOEC.html

9
http://preprocessed-connectomes-project.org/

10
http://preprocessed-connectomes-project.org/abide/Pipelines.html
